# The C-terminal tail of Cf resistance proteins determines the intensity of the effector-triggered hypersensitive response-related cell death

**DOI:** 10.1093/plphys/kiag476

**Published:** 2026-07-31

**Authors:** Esranur Budak, Lisa van Malssen, Sergio Landeo Villanueva, Sjef Boeren, İkbal Agah İnce, Edouard Evangelisti, Matthieu H A J Joosten

**Affiliations:** Laboratory of Phytopathology, Department of Plant Sciences, Droevendaalsesteeg 1, 6708 PB Wageningen, The Netherlands; Laboratory of Phytopathology, Department of Plant Sciences, Droevendaalsesteeg 1, 6708 PB Wageningen, The Netherlands; Laboratory of Phytopathology, Department of Plant Sciences, Droevendaalsesteeg 1, 6708 PB Wageningen, The Netherlands; Laboratory of Biochemistry, Wageningen University, Stippeneng 4, 6708 WE Wageningen, The Netherlands; Department of Pathology and Medical Biology, University of Groningen, University Medical Center Groningen, Hanzeplein 1, 9713 GZ Groningen, The Netherlands; Centre de Biologie Structurale, CNRS UMR 5048-UM-INSERM U 1054, 29 rue de Navacelles 34090, Montpellier, France; Laboratory of Phytopathology, Department of Plant Sciences, Droevendaalsesteeg 1, 6708 PB Wageningen, The Netherlands; Laboratory of Phytopathology, Department of Plant Sciences, Droevendaalsesteeg 1, 6708 PB Wageningen, The Netherlands

## Abstract

Cf resistance proteins of tomato that provide resistance against the extracellular fungal pathogen *Fulvia fulva* are receptor-like proteins (RLPs) localized at the cell surface. As they lack a cytoplasmic kinase domain, Cf proteins require 2 co-receptors to activate downstream immune responses. In the resting state, Cf proteins constitutively interact with the receptor-like kinase (RLK) SUPPRESSOR OF BIR1-1 (SOBIR1), whereas upon recognition of the matching avirulence (Avr) effector, the RLK BRI1-ASSOCIATED KINASE 1 (BAK1) is recruited. Cf proteins have a typical leucine-rich repeat (LRR)-RLP structure, consisting of an LRR ectodomain, a transmembrane domain, and an intracellular juxtamembrane domain that is referred to as the C-terminal tail. Cf-4 and Cf-9 have identical C-terminal tails, while the C-terminal tails of Cf-2 and Cf-5 differ by a single amino acid. Interestingly, the Cf-5/Avr5- and Cf-2/Avr2-triggered immune response results in a slower and weaker hypersensitive response (HR)-related cell death than the response triggered by Cf-4/Avr4 and Cf-9/Avr9. Domain swapping between Cf-5 and Cf-9 revealed that the C-terminal tail plays a specific role in determining the intensity of the immune response. Notably, the pool of SOBIR1-associated Cf-4 and Cf-9 proteins is much larger than that of Cf-2 and Cf-5, and a full-length C-terminal tail is required for immune signaling activation. This work provides a basis for further studies on RLP engineering to enhance crop resistance against detrimental pathogens.

## Introduction

Plants have evolved a 2-layered innate immunity system to protect themselves against pathogens (Dodds and Rathjen 2010). The first layer is mediated by cell surface receptors that recognize extracellular immunogenic patterns, such as microbe-associated molecular patterns (MAMPs) or microbial extracellular effectors. These compounds trigger the initiation of downstream signaling, which eventually leads to extracellularly triggered immunity (van der Burgh and Joosten 2019). Cell surface receptors that are localized at the plasma membrane (PM) can be divided into 2 groups, receptor-like kinases (RLKs) and receptor-like proteins (RLPs) (Macho and Zipfel 2014). RLPs and RLKs share structural similarity, such as the presence of a ligand-binding ectodomain (ECD), for which the leucine-rich repeat (LRR) is one of the major motifs, and which is involved in the perception of proteinaceous ligands, and a transmembrane (TM) domain. In addition, RLKs have a cytoplasmic protein kinase domain (KD), while RLPs lack such a domain (Monaghan and Zipfel 2012; Liebrand et al. 2013; Zhang et al. 2013a; Böhm et al. 2014; Zipfel 2014; Ranf 2017).

LRR-RLPs play an important role in plant immunity. Cf proteins, which are tomato (*Solanum lycopersicum*, *Sl*) resistance proteins against the pathogenic fungus *Fulvia fulva,* formerly known as *Cladosporium fulvum*, are well-known LRR-RLPs playing a role in dominant gene-for-gene resistance, and perceive avirulence (Avr) effectors secreted by *F. fulva*. Cf-4, Cf-9, Cf-5, and Cf-2 are examples of identified Cf proteins, which recognize the matching *F. fulva* effectors Avr4, Avr9, Avr5, and Avr2, respectively (Dixon et al. 1996, 1998; Jones et al. 1994; Thomas et al. 1997) In addition, the tomato LRR-RLP Ve1 is a resistance protein against *Verticillium* spp., secreting the matching Ave1 effector (Kawchuk et al. 2001). Furthermore, RLP23 is an *Arabidopsis thaliana* LRR-RLP that perceives necrosis- and ethylene-inducing-like proteins (NLPs) from fungi, bacteria, and oomycetes (Albert et al. 2015). RLP42, which is another LRR-RLP from *A. thaliana*, recognizes endo polygalacturonases (PGs) from bacteria (Zhang et al. 2014), whereas RESPONSE TO XEG1 (RXEG1), an LRR-RLP from *Nicotiana benthamiana*, recognizes XEG1, which is a xyloglucanase effector that *Phytophthora sojae* secretes into the apoplast (Wang et al. 2018).

LRR-RLPs constitutively interact with the co-receptor SUPPRESSOR OF BIR1-1/EVERSHED (SOBIR1/EVR), which compensates for their lack of a KD. SOBIR1 was identified as a co-purifying protein upon pull-down of a Cf-4-eGFP fusion and appeared to be essential for the function of all LRR-RLPs involved in plant immunity that have been studied up till now (Liebrand et al. 2013). Upon perception of the matching ligand, the LRR-RLK SOMATIC EMBRYOGENESIS RECEPTOR KINASE 3/BRI1-ASSOCIATED RECEPTOR KINASE 1 (SERK3/BAK1) is recruited by the LRR-RLP/SOBIR1 complex and trans-phosphorylation events between the cytoplasmic KDs of SOBIR1 and BAK1 initiate downstream immune signaling, followed by an apoplastic reactive oxygen species (ROS) burst, activation of mitogen-activated protein kinases (MAPKs), and transcriptional regulation, and finally leading to hypersensitive response (HR)-related cell death, although such a type of cell death does not occur for all LRR-RLP/SOBIR1 complexes (Liebrand et al. 2014; Albert et al. 2015; Postma et al. 2016; van der Burgh et al. 2019). Studies on Cf-4 revealed that immune signaling downstream of Cf-4 is differentially regulated by different receptor-like cytoplasmic kinases (RLCKs), especially RLCKs from subfamilies VII-6, VII-7, and VII-8 (Huang et al. 2024b). Moreover, the network of helper nucleotide-binding and leucine-rich repeat (NLR) proteins required for cell death (NRCs) differentially regulates the initiation of the Cf-4-triggered HR-related cell death. For example, the helper NLR NRC3 is a core protein required for cell death, whereas NRC4 does not contribute to this process (Kourelis et al. 2022).

The extracellular LRR motif of LRR-RLPs is interrupted by a non-LRR part, referred to as the island domain or loop-out region, and which is present close to the C-terminus of the LRR domain (Fritz-Laylin et al. 2005; Snoeck et al. 2023). Recently, the crystal structure of RXEG1 revealed that upon recognition of XEG1, conformational changes occur in the island domain of RXEG1, enabling its interaction with BAK1. Additionally, the C-terminal 4 LRRs of RXEG1, just above the TM domain, directly bind to the 5 LRRs of BAK1 (Sun et al. 2022). BAK1 recruitment is essential for the function of many LRR-RLPs involved in immunity, and since an island domain is present in most of these receptors, probably the island domain of LRR-RLPs plays a conserved role in ligand-induced BAK1/LRR-RLP interaction (Fradin et al. 2009; Bar et al. 2010; Liebrand et al. 2014; Zhang et al. 2021).

The TM domain of LRR-RLPs frequently contains a GxxxG (G, glycine; x, any amino acid) motif that is either single or tandemly arranged. SOBIR1 contains a double GxxxG motif in its TM domain, which is referred to as a glycine zipper (GxxxGxxxG) motif (Gust and Felix 2014). It has been shown that the GxxxGxxxG motif of SOBIR1 is essential for its interaction with the Cf-4 protein (Bi et al. 2016). LRR-RLPs only have a short intracellular juxtamembrane (iJM) domain, also referred to as the C-terminal tail, of which the function is unknown. However, the expression of a truncated form of RXEG1 lacking its C-terminal tail, in an *RXEG1*-knockout of *N. benthamiana*, failed to trigger cell death upon challenge with XEG1 (Sun et al. 2022). In another study, the C-terminal tail of Ve1 was deleted or swapped with the C-terminal tail of Ve2. Both Ve1 lacking its C-terminal tail and Ve1 containing the C-terminal tail of Ve2 were unable to trigger cell death when challenged with Ave1 (Fradin et al. 2014). These studies suggest that the C-terminal tail of RXEG1 and Ve1 is required for their function. In contrast, RLP23 lacking its C-terminal tail showed a reduction in the intensity of the immune response that is triggered upon perception of nlp20, but did not completely lose its functionality (Albert et al. 2019). In another study, the KD and TM domain of the RLK ELONGATION FACTOR-TU RECEPTOR (EFR), which recognizes the elongation factor-Tu peptide elf18, were replaced by the TM domain and C-terminal tail of Cf-9. Interestingly, the resulting EFR/Cf-9 chimeric protein still recognizes elf18, but now triggers a strong SOBIR1-dependent HR-related cell death, similar to the strong response that is triggered by the Cf-9/Avr9 combination. EFR does not trigger cell death upon recognition of the elf18 peptide (Wu et al. 2019).

Even though all LRR-RLPs involved in immunity appear to interact with the same co-receptors, SOBIR1 and BAK1, their immune output is different. For example, the BAK1/RLP42/SOBIR1 complex triggers HR-related cell death upon ligand recognition, whereas the BAK1/RLP23/SOBIR1 complex does not (Albert et al. 2015; Zhang et al. 2021). Ve1 and Ve2 share 87% similarity in their amino acid sequences; however, only Ve1 confers resistance to *Verticillium dahliae*, whereas Ve2 does not. Both receptors have been shown to interact with SOBIR1 (Kawchuk et al. 2001; Fradin et al. 2014). Additionally, Ve1 interacts with BAK1 (Fradin et al. 2009), but there is currently no evidence that Ve2 also interacts with BAK1, although a possible interaction between Ve2 and BAK1 has been suggested to take place (Kalischuk et al. 2022). Notably, only Ve1 triggers HR-related cell death upon recognition of Ave1, while Ve2 does not trigger such a response (De Jonge et al. 2012). It has been shown that the C-terminal tail of Ve2, which is much longer than that of Ve1, is unable to trigger HR-related cell death (Fradin et al. 2014). Moreover, the Cf-4 and Cf-9 proteins trigger a very strong and fast cell death, visible as collapsed mesophyll tissue, upon leaf infiltration with their matching effector proteins Avr4 and Avr9, respectively (van der Hoorn et al. 2000; Cai et al. 2001). On the other hand, the Cf-2/5-triggered response is less strong, only resulting in chlorosis, and its development takes several days after infiltration (Brading et al. 2000). Interestingly, the C-terminal tails of Cf-4 and Cf-9 are identical, and the C-terminal tails of Cf-2 and Cf-5 also share high similarity (Bi et al. 2016). The C-terminal tail of the Cf proteins is the only domain of these LRR-RLPs that is present in the cytoplasm of the cell, and whether this domain has any role in determining the intensity of the HR-related cell death is currently unknown.

In this study, we focused on the possible role of the C-terminal tail of Cf proteins in modulating the intensity of the immune response. To dissect whether and how the C-terminal tail contributes to the immune output, we generated various Cf-5-chimeric proteins containing the TM domain and/or the C-terminal tail of Cf-4, and Cf-4-chimeric proteins containing the TM domain and/or the C-terminal tail of Cf-5. Interestingly, upon challenge with the matching Avr protein, the Cf-5-chimeric proteins containing the C-terminal tail of Cf-4 triggered a stronger HR-related cell death when compared to the Cf-4-chimeric proteins containing the Cf-5 C-terminal tail. In fact, the Cf-5/4-chimeric proteins triggered a response as strong as Cf-4 and Cf-9, whereas the Cf-4/5-chimeric proteins triggered a response resembling that of Cf-2 and Cf-5. We observed that the C-terminal tail of Cf proteins has a role in determining the intensity of the HR-related cell death, in contrast to the TM domain of the Cf proteins. We did not find evidence for the C-terminal tail to have a specific role in modulating the intensity of the apoplastic ROS burst or MAPK activation. Moreover, the immune output of Cf-chimeric proteins containing the C-terminal tail of Ve1 or Ve2 suggests that Cf proteins need a C-terminal tail that is able to trigger cell death. Deletion of the C-terminal tail of Cf-4 indicated that the presence of a full-length C-terminal tail is essential for a proper Cf-4/Avr4-triggered immune response. Furthermore, our data show that the Cf-4 and Cf-9 proteins have a stronger interaction with SOBIR1 than the Cf-2 and Cf-5 proteins, as the pool of SOBIR1-associated Cf protein is much larger for Cf-4 and Cf-9, when compared to that of Cf-2 and Cf-5. It appears that the TM domain and C-terminal tail of the Cf proteins play a role in the strength by which SOBIR1 is recruited.

## Results

### Cf-4/9 and Cf-2/5 have different C-terminal tails and trigger HR-related cell death of different intensity

**Figure 1:**
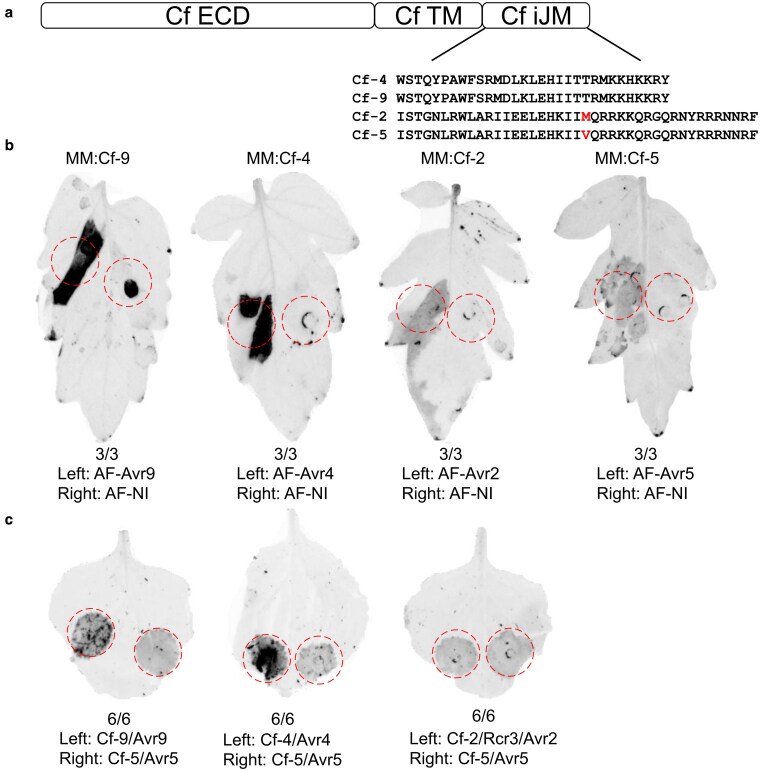
The C-terminal tails of Cf-4 and Cf-9 are identical, and both Cf proteins trigger a strong HR-related cell death, whereas Cf-2 and Cf-5, which also have very similar C-terminal tails, trigger a less strong HR-related cell death. a) Schematic representation of Cf protein structure. ECD, ectodomain; TM, transmembrane; iJM, intracellular juxtamembrane domain. A sequence alignment of the iJM domains of Cf-9, Cf-4, Cf-2, and Cf-5 proteins is shown, with the 1 amino acid residue difference between Cf-5 and Cf-2 indicated in red. b) Leaves of tomato cultivar Moneymaker (MM) Cf-9, MM:Cf-4, MM:Cf-2, and MM-Cf-5 were infiltrated with AF containing the matching *F. fulva* effectors Avr9, Avr4, Avr2, and Avr5, respectively. AF from noninoculated plants (AF-NI) was used as a control. The images were taken under red light at 5 dpi, except for MM:Cf-9, of which a picture was taken at 2 dpi. c) In the left half of the leaves, Cf-9, Cf-4, and Cf-2 were transiently co-expressed with Avr9, Avr4, and Avr2 + Rcr3, respectively. For comparison of the cell death intensities, in each right half of the leaves, the Cf-5/Avr5 combination was transiently expressed. Pictures were taken at 7 dpi, under red light. For tomato *n* = 3, and for *N. benthamiana n* = 6. The experiments were repeated 3 times with similar results, and representative images are shown.

Cf-4 and Cf-9 have an identical C-terminal tail, and similarly, the C-terminal tails of Cf-2 and Cf-5 also share very high resemblance, as the difference is only 1 amino acid residue (Fig. 1a) (Thomas et al. 1998). Interestingly, upon recognition of their matching effector, Cf-4 and Cf-9 trigger stronger HR-related cell death when compared to Cf-2 and Cf-5 (van der Hoorn et al. 2000; Rooney et al. 2005). Leaves of tomato plants expressing Cf-9, Cf-4, Cf-2, and Cf-5 in the background of cultivar Moneymaker (MM) were infiltrated with apoplastic fluid (AF) containing their corresponding effectors, Avr9, Avr4, Avr2, and Avr5, respectively, isolated from *F. fulva*-infected tomato leaves. As a negative control, leaves were infiltrated with AF from noninfected tomato plants. Cf-9/Avr9-infiltrated leaves were sampled at an earlier time point due to the rapid onset of the immune response. At 5 d post-infiltration (dpi), the HR-related cell death triggered by Cf-9/Avr9 and Cf-4/Avr4 was markedly stronger than the response induced by Cf-2/Avr2 and Cf-5/Avr5. AF from noninfected plants did not trigger cell death in any of the tomato lines (Fig. 1b). Cf-2 requires the extracellular tomato protease REQUIRED FOR *CLADOSPORIUM* RESISTANCE 3 (RCR3) for its functionality (Rooney et al. 2005), which is lacking in *N. benthamiana* and therefore has to be co-expressed. The Cf-9/Avr9, Cf-4/Avr4, Cf-2/Avr2/Rcr3, and Cf-5/Avr5 combinations were transiently expressed by agroinfiltration in leaves of *N. benthamiana*, and at 5 dpi, a much stronger HR-related cell death was observed with Cf-9/Avr9 and Cf-4/Avr4, when compared to Cf-2/Avr2/Rcr3 Cf-5/Avr5. None of the Cf proteins triggered cell death when co-expressed with nonmatching effectors in *N. benthamiana* (Figure S1). Overall, Cf-4 and Cf-9 trigger a more intense and faster cell death than Cf-2 and Cf-5, also in a heterologous system (Fig. 1c). This points out that the C-terminal tail of the Cf proteins might be involved in determining the intensity of the HR-related cell death.

### Cf-5-chimeric constructs containing the C-terminal tail of Cf-4 trigger a stronger HR-related cell death than Cf-5 itself

**Figure 2:**
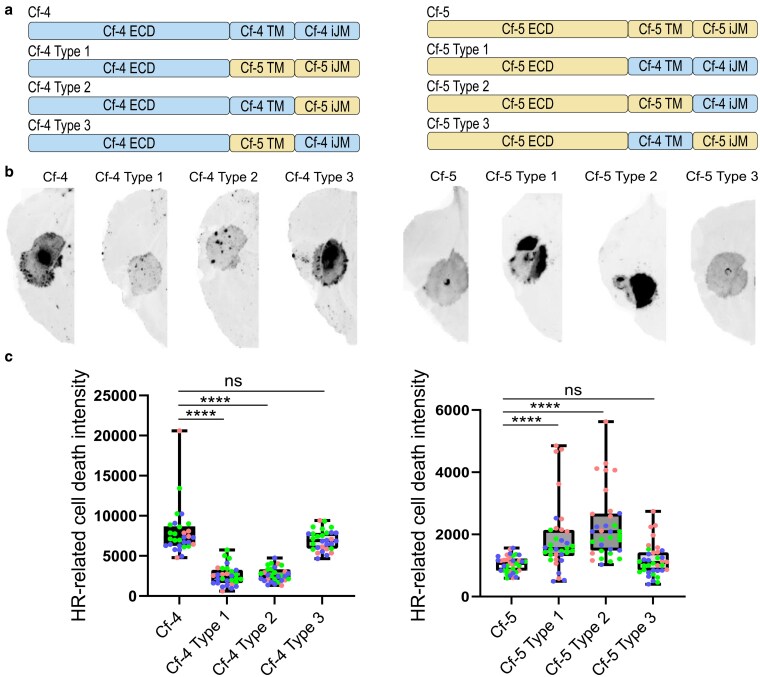
Chimeric Cf proteins containing the C-terminal tail of Cf-4 trigger a strong HR-related cell death. a) Overview of the domain structure of the chimeric receptors that have been generated. The domains originating from the Cf-4 and Cf-5 proteins are indicated in blue and yellow, respectively. ECD, ectodomain; TM, transmembrane; iJM, intracellular juxtamembrane domain. b and c) The chimeric Cf-4 and Cf-5 constructs, all C-terminally fused to eGFP, were transiently co-expressed with their matching effector in leaves of *N. benthamiana* (OD_600_ = 0.8). In (b), in the 4 leaf halves shown on the left, Cf-4 and the 3 chimeric Cf-4 proteins were transiently expressed, whereas in the 4 leaf halves shown on the right Cf-5 and the 3 chimeric Cf-5 proteins were transiently expressed. The leaves were imaged under red light, at 6 dpi. c) The intensity of the HR-related cell death triggered as shown in (b) was quantified by Image Lab. The different experimental replicates are indicated with different colors. A 1-way ANOVA/Dunnett’s multiple comparison test was used to determine statistical significance. Central line, median; error bars, min–max, *n* = 12. ****P < 0.0001; ns, nonsignificant; *P* > 0.05.

To investigate the role of the C-terminal tail of the Cf proteins in determining the intensity of the HR-related cell death, we generated 6 chimeric Cf proteins (Fig. 2a). Cf-4, Cf-5, and all chimeric Cf proteins, all fused to eGFP at their C-terminus, were transiently co-expressed by agroinfiltration with their matching effector in *N. benthamiana*, and the response that was triggered was imaged at 6 dpi.

Interestingly, the HR-related cell death triggered by the chimeric Cf-5 proteins carrying the C-terminal tail of Cf-4 (Cf-5 Type 1 and Cf-5 Type 2) was markedly stronger, comparable in intensity to the response induced by Cf-4/Avr4 (Fig. 2b). In contrast, the chimeric Cf-5 construct containing only the TM domain of Cf-4 (Cf-5 Type 3) elicited a weaker HR-related cell death. Similarly, chimeric Cf-4 proteins containing the C-terminal tail of Cf-5 (Cf-4 Type 1 and Cf-4 Type 2) triggered a weaker response, which is very similar to Cf-5/Avr5-triggered HR-related cell death. However, a stronger response was observed when the chimeric Cf-4 protein containing only the TM domain of Cf-5 (Cf-4 Type 3) was transiently co-expressed with Avr4 (Fig. 2b).

The intensity of the HR-related cell death triggered by Cf-4, Cf-5 and the various chimeric Cf proteins was quantified by red light imaging (Landeo Villanueva et al. 2021) and it was clear that the intensity of the response triggered by the chimeric Cf-5 proteins containing the C-terminal tail of Cf-4 (Cf-5 Type 1 and Type 2) was much higher than the intensity of the HR-related cell death triggered by Cf-5 itself (Fig. 2c). The chimeric Cf-5 protein containing only the TM domain of Cf-4 (Cf-5 Type 3), triggered a response with an intensity similar to that triggered by Cf-5 itself. Interestingly, the results for the chimeric Cf-4 proteins were compatible with the results obtained for the Cf-5-chimeric proteins, as the chimeric Cf-4 proteins containing the C-terminal tail of Cf-5 (Cf-4 Type 1 and Type 2) triggered a less strong cell death than Cf-4 itself. However, the intensity of the response triggered by the chimeric Cf-4 protein only containing the TM domain of Cf-5 (Cf-4 Type 3) was similar to that of Cf-4 itself (Fig. 2c). The immunoprecipitation results indicate that all Cf proteins and chimeric variants accumulate at broadly similar levels following GFP immunoprecipitation (Figure S2). However, because the proteins are not detectable in the input samples, possible differences in protein accumulation levels can also contribute to the observed phenotypes. By domain swaps with Cf-2, we show that the single amino acid residue difference between Cf-2 and Cf-5 in their C-terminal tail does not affect the intensity of the cell death response (Figure S3). Furthermore, the strong responses triggered by Cf-4 Type 3/Avr4, Cf-5 Type 1/Avr5, and by Cf-5 Type 2/Avr5 all remain dependent on NRC2/3, similar to the HR-related cell death induced by the Cf-4/Avr4 combination itself (Figure S4). These results demonstrate that all chimeric Cf proteins containing the C-terminal tail of the Cf-5 protein trigger relatively weak HR-related cell death symptoms. On the other hand, a strong response is triggered by all chimeric Cf proteins containing the C-terminal tail of Cf-4/9. Moreover, the presence of the TM domain of either Cf-4 or Cf-5 does not affect the intensity of the cell death. To summarize, the C-terminal tail of the various Cf proteins has a specific role in determining the intensity of the HR-related cell death, while the TM domainof the Cf proteins does not.

**Figure 3:**
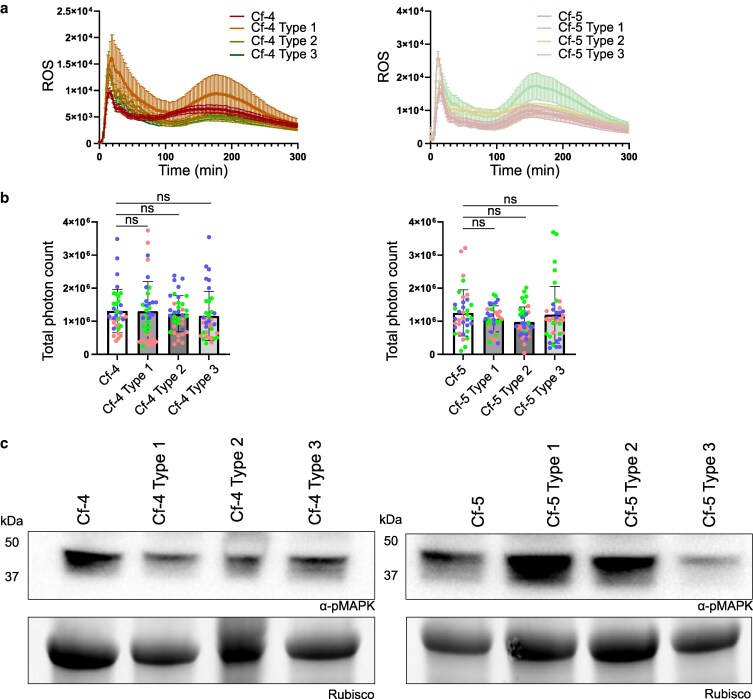
Chimeric Cf proteins containing the C-terminal tail of Cf-4 do not trigger a stronger ROS burst or activate stronger MAPK signaling, when compared to those containing the C-terminal tail of Cf-5. a) Chimeric Cf-4 and Cf-5 constructs were expressed by agroinfiltration in leaves of *N. benthamiana.* At 2 dpi, leaf discs were taken and incubated with their matching effector at a concentration of 0.1 µM, during which apoplastic ROS production was measured by a luminol-based assay over a period of 5 h (*n* = 12). Error bars indicate the standard error. The experiment was repeated at least 3 times, and a representative result is shown. b) Total photon counts of the ROS bursts shown in (a) were calculated over a period of 5 h. The experiment was repeated at least 3 times, with each independent replicate indicated by a different color. An ANOVA/Dunnett's multiple comparison test was used to determine statistical significance. Error bars represent SD, *n* = 12. ****P < 0.0001; ns, nonsignificant. c) Chimeric Cf-4 and Cf-5 proteins were expressed by agroinfiltration in leaves of *N. benthamiana.* At 2 dpi, leaves were infiltrated with the matching effectors, and after 15 min total proteins were extracted and subjected to immunoblotting, followed by incubation of the blot with p42/p44-erk antibody to detect activated MAPKs. Rubisco bands indicate the amount of total protein loading. The experiment was repeated at least 2 times, and a representative image is shown.

In addition, apoplastic ROS and MAPK activation assays, which are typical read-outs of the immune response, were performed. For this, we transiently expressed all 8 proteins as shown in Fig. 2a in leaves of *N. benthamiana* to first investigate the role of the C-terminal tail of the Cf proteins in the intensity of the apoplastic ROS burst. Leaf discs were taken at 2 dpi and the discs were incubated with the Avr4 or Avr5 effector over a period of 5 h. Cf-4 and its chimeric variants all triggered a clear biphasic ROS burst upon elicitation with Avr4 (Fig. 3a, left panel). The total photon counts, used to quantify the overall intensity of the apoplastic ROS burst, revealed no significant differences between the ROS generated by Cf-4 itself and the 3 chimeric Cf-4 proteins (Fig. 3b, left panel). Similarly, Cf-5 and its chimeric variants all triggered a clear biphasic ROS burst upon incubation with Avr5, and the total photon counts also showed no significant differences between the ROS generated by Cf-5 and the chimeric Cf-5 proteins (Fig. 3a and 3b, right panels). For determining MAPK activation, all constructs were once more expressed by agroinfiltration in leaves of *N. benthamiana*, after which at 2 dpi the leaves expressing Cf-4 and its chimeric variants were infiltrated with Avr4 protein, while those expressing Cf-5 and its chimeric variants were infiltrated with Avr5 protein and incubated for 15 min. Then SDS–PAGE, followed by western blotting was performed to determine the level of MAPK activation, using an antibody raised against phosphorylated MAPKs. No notable differences in MAPK activation as detected by the anti-p42/p44-ERK antibody were observed at 15 min when the different chimeric Cf proteins were compared (Fig. 3c). Overall, these results indicate that the C-terminal tail of the Cf proteins does not play a role in determining the intensity of the apoplastic ROS burst and that the C-terminal tail is also not affecting the level of activation of the MAPKs as detected by the anti-p42/p44-ERK antibody at the 15 min time point.

### Cf proteins need a C-terminal tail that is able to trigger cell death and a biphasic ROS burst

Ve1 and Ve2 are RLPs that share 84% amino acid identity (Kawchuk et al. 2001). The Ave1 effector of *V. dahliae* is recognized by both Ve1 and Ve2, and upon recognition of Ave1 by Ve1, an HR-related cell death is triggered, whereas upon recognition by Ve2, cell death does not take place (Zhang et al. 2013b). Fradin et al. (2014) showed that chimeric Ve1 proteins containing the C-terminal tail of Ve2, which is much longer than that of Ve1, do not trigger cell death upon challenge with Ave1 in *Nicotiana tabacum.* Therefore, they concluded that the C-terminal tail of Ve2 is not able to trigger cell death (Fradin et al. 2014).

**Figure 4:**
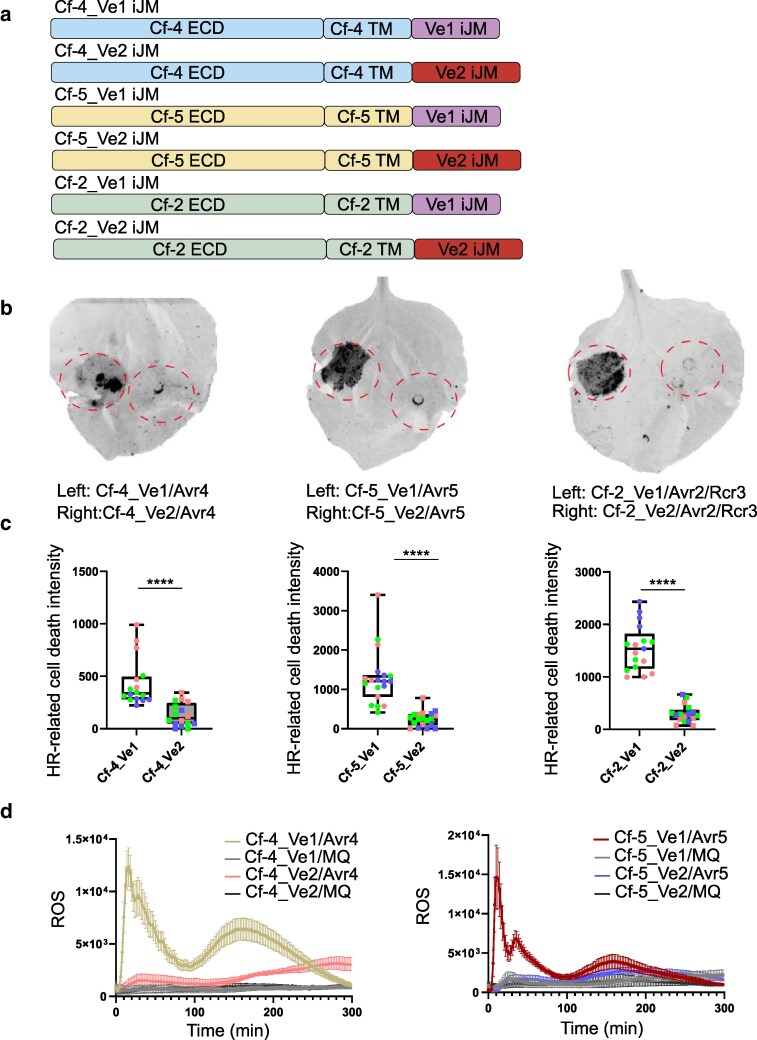
Cf proteins containing the C-terminal tail of Ve1 respond to their matching effectors and trigger both HR-related cell death and a biphasic ROS burst, whereas this is not the case when their C-terminal tail is replaced by that of Ve2. a) Overview of the domain structure of the chimeric Cf receptors that have been generated. The domains originating from the Cf-4, Cf-5, and Cf-2 proteins are indicated in blue, yellow, and green, respectively. The attached C-terminal tails of Ve1 and Ve2 are indicated in purple and red, respectively. b) The chimeric constructs were transiently co-expressed with their matching effector in leaves of *N. benthamiana* (OD_600_ = 0.8), after which they were imaged at 6 dpi under red light. c) The intensity of the HR-related cell death as shown in (b) was quantified by Image Lab, and a Student's *t*-test was used to determine statistical significance. The experiment was repeated at least 3 times, with each independent replicate indicated by a different color. Central line, median; error bars, min–max, *n* = 5 to 6. Significance between groups (unpaired 2-tailed *t*-test, *P* < 0.05) is indicated with an asterisk, ****P < 0.0001. d) Chimeric Cf-4 and Cf-5 constructs were transiently expressed in leaves of *N. benthamiana* (OD_600_ = 0.1), and at 2 dpi, leaf discs were taken and incubated with 0.1 µM Avr4 and 0.1 µM Avr5 protein, respectively, after which ROS production was measured by a luminol-based assay over a period of 5 h (*n* = 12). Error bars indicate the standard error. Experiments were repeated 3 times, with similar results. Representative experiments are shown.

To investigate whether Cf proteins need a C-terminal tail that is able to trigger HR-related cell death, we generated 6 chimeric Cf proteins containing the C-terminal tail of either Ve1 or Ve2 (Fig. 4a). The chimeric Cf proteins were all transiently co-expressed with their matching effectors in leaves of *N. benthamiana*, and at 6 dpi, the cell death that was triggered was imaged by red light and subsequently quantified. The chimeric Cf proteins containing the C-terminal tail of Ve1 all triggered HR-related cell death upon challenge with their matching effector, whereas the chimeric Cf proteins containing the C-terminal tail of Ve2 failed to trigger cell death (Fig. 4b and 4c).

Next, these chimeric Cf proteins were transiently expressed in leaves of *N. benthamiana* to determine the intensity of the apoplastic ROS burst upon incubation with their matching effectors. At 2 dpi, the leaf discs were incubated with the matching effector over a period of 5 h, and the addition of milli-Q water (MQ) was used as a negative control. The chimeric Cf proteins containing the C-terminal tail of Ve1 triggered a typical biphasic ROS burst upon incubation with their matching effector. On the other hand, the typical biphasic ROS burst was not observed when leaf discs of *N. benthamiana* transiently expressing the chimeric proteins containing the C-terminal tail of Ve2 were challenged with their matching effector. As expected, treatment with MQ did not result in a ROS burst (Fig. 4d).

Cf proteins require interaction with both SOBIR1 and BAK1 to trigger an immune response (van der Burgh et al. 2019). Therefore, in addition to assessing their proper accumulation, the interaction of the chimeric Cf proteins with SOBIR1 and BAK1 was also investigated. The various chimeric proteins were properly detected following GFP immunoprecipitation in *N. benthamiana*, although the Cf proteins containing the C-terminal tail of Ve2 generally showed a weaker signal than those containing the Ve1 C-terminal tail (Figure S5a and S5b). In addition, a co-IP of the chimeric Cf proteins with *Sl*SOBIR1 and *Sl*BAK1 showed that all chimeric proteins interact with both SOBIR1 and BAK1 (Figure S5a and S5b), indicating that the C-terminal tail of Ve1 or Ve2 does not affect the interaction of the chimeric Cf proteins with SOBIR1 and BAK1.

### Cf-4 needs its entire C-terminal tail for triggering fast, confluent cell death

**Figure 5:**
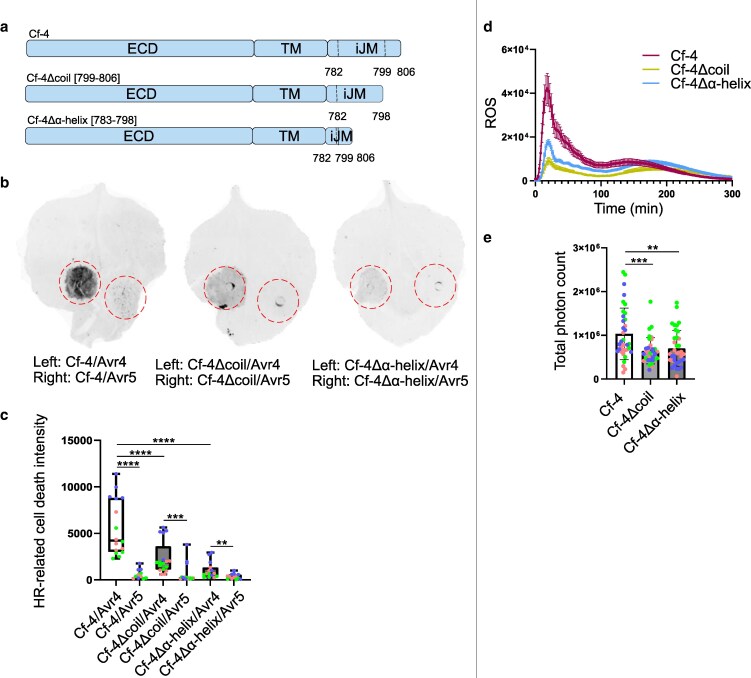
Cf-4 requires its full-length C-terminal tail to trigger confluent cell death and a strong biphasic ROS burst. a) Overview of the domain structure of the Cf-4 protein and the Cf-4 deletion constructs of the last coil and the α-helix of the C-terminal tail. The various domains of the Cf-4 protein are indicated in blue. b) Cf-4, the Cf-4 protein of which the last coil of the C-terminal tail was deleted (Cf-4Δcoil) and the Cf-4 protein of which the α-helix of the C-terminal tail was deleted (Cf-4Δα-helix), were transiently co-expressed with the matching effector Avr4 in *N. benthamiana* (OD_600_ = 0.8). The leaves were imaged at 6 dpi under red light. c) The intensity of the HR-related cell death was quantified by Image Lab, and a Student's *t*-test was used to determine statistical significance. Central line, median; error bars, min–max, *n* = 5 to 6. Significance between groups (unpaired 2-tailed *t*-test, *P* < 0.05) is indicated with an asterisk, ****P < 0.0001, ***P < 0.001, **P < 0.01. d) Cf-4, Cf-4Δcoil, and Cf-4Δα-helix were transiently expressed in *N. benthamiana,* and at 2 dpi, after which leaf discs were taken and incubated with 0.1 µM Avr4 protein during which ROS production was measured over a period of 5 h by a luminol-based assay (*n* = 12). The bars indicate the standard error. The experiment was repeated 3 times, and a representative image is shown. c and d) The experiments were repeated 3 times, with each independent replicate indicated by a different color. e) Total photon counts were calculated over a period of 5 h. An ANOVA/Dunnett's multiple comparison was used to determine statistical significance. Error bars display the SD. ****P < 0.0001, ***P < 0.001, **P < 0.01.

The secondary structure of the C-terminal tail of Cf-4/9, Cf-2, Cf-5, Ve1, and Ve2 was predicted by using the online tool NetSurfP-3.0 and AlphaFold. The C-terminal tail of Cf-4/9, Cf-2, Cf-5, and Ve1 is predicted to contain 2 regions with a nonregular secondary structure, referred to as coils, 1 at the N-terminal part and 1 at the C-terminal end of the tail, with 1 α-helix in the middle. The C-terminal tail of Ve2 is much longer and has an additional, different secondary structure, as it contains coils, an α-helix, and additional β-sheets (Figure S6). We performed a truncation analysis on the C-terminal tail of Cf-4 to investigate the requirements of the coil and α-helix domains for the initiation of downstream immune responses. Two deletions were introduced at the C-terminal end of Cf-4; the first deletion concerned the last coil (Cf-4Δcoil) and the second deletion concerned the α-helix domain (Cf-4Δα-helix) (Fig. 5a). The constructs were transiently expressed in leaves of *N. benthamiana*, in combination with the Avr4 effector, and the HR-related cell death that was triggered was imaged. Cf-4/Avr4 expression resulted in collapsed tissue, while Cf-4Δcoil and Cf-4Δα-helix expression, in combination with Avr4, did not result in a clear cell death response, as shown in Fig. 5b. The intensity of the HR-related cell death triggered by the various constructs was quantified by red light imaging, which revealed that the intensity of the Cf-4-triggered cell death was significantly stronger than the cell death triggered by the deletion constructs (Fig. 5c). Furthermore, leaf discs were taken from the leaves that were expressing the different constructs and incubated with the Avr4 effector over a period of 5 h. A typical biphasic apoplastic ROS burst was observed in all cases, but the total photon counts revealed that the truncations in the C-terminal tail of Cf-4 lowered the intensity of the ROS burst (Fig. 5d and 5e). Note that all 3 proteins were detected at similar levels after GPF immunoprecipitation. (Figure S7).

### The amount of SOBIR1 protein that co-purifies with Cf-4 and Cf-9 is consistently larger than the amount co-purifying with Cf-2 and Cf-5

**Figure 6:**
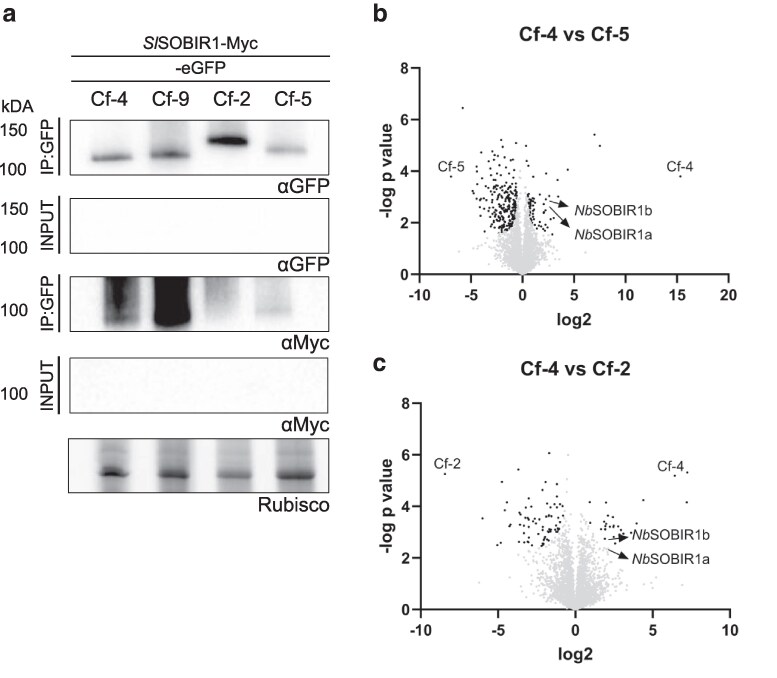
The pool of Cf-4 and Cf-9 proteins interacting with SOBIR1 is larger than that of Cf-2 and Cf-5. (a**)** Cf-4, Cf-9, Cf-2, and Cf-5, all tagged with eGFP, were co-expressed with *Sl*SOBIR1-Myc in leaves of *N. benthamiana*. After 2 dpi, total protein was extracted and incubated with GFP beads for immunoprecipitation. The immunoprecipitated proteins were subjected to immunoblotting with αGFP antibodies to visualize the eGFP-tagged Cf proteins and with αMyc antibodies to detect the Myc-tagged SOBIR1 protein. Rubisco bands indicate the total protein content of the input samples. b and c) Cf-4-eGFP, Cf-5-eGFP, and Cf-2-eGFP were transiently expressed in leaves of *N. benthamiana*, and at 2 dpi, total proteins were extracted and subjected to immunoprecipitation using GFP beads. Peptides were obtained from the immunoprecipitated proteins by a tryptic digest and were analyzed by LFQ nano-MS/MS. b) Comparison of the co-immunopurified proteins between Cf-4 and Cf-5, and c) between Cf-4 and Cf-2, by volcano plots. Significantly enriched proteins are indicated by the black dots. The light gray dots represent proteins that are not significantly enriched. Note that with Cf-4-eGFP, more SOBIR1 protein is purified than with Cf-5-eGFP or Cf-2-eGFP.

Cf proteins constitutively interact with their co-receptor SOBIR1. It has been shown before that the amount of SOBIR1 co-purifying with Cf-9 appeared to be substantially larger than with Cf-2 (Liebrand et al. 2013). Notably, Cf-9 triggers a much stronger HR-related cell death than Cf-2. To further investigate whether Cf-4 and Cf-9, which both trigger strong cell death, consistently interact with larger amounts of SOBIR1 protein than Cf-5 and Cf-2, which trigger a less strong cell death, we performed co-immunoprecipitation assays between SOBIR1 and Cf-4, Cf-9, Cf-5, and Cf-2. The results indicated that the amount of SOBIR1 protein interacting with the pulled-down Cf-4 and Cf-9 proteins was indeed much larger than the amount of SOBIR1 protein interacting with the Cf-5 and Cf-2 proteins (Fig. 6a). This observation indicates that the overall pool of accumulating Cf protein actually interacting with SOBIR1 is much larger for Cf-4/9 than for Cf-5/2.

Moreover, we further tested this co-immunoprecipitation result by mass spectrometry analysis. For this, Cf-4, Cf-5, and Cf-2, all tagged with eGFP, were transiently expressed in leaves of *N. benthamiana*, and at 2 dpi, total protein was extracted, and immunoprecipitation of the Cf proteins was again performed by GFP beads. The immunoprecipitated proteins were subsequently on-bead digested by trypsin and the resulting peptides were analyzed by mass spectrometry, followed by label-free quantification (LFQ). Volcano plots were made to compare the co-purifying proteins between Cf-4 and Cf-5, as well as between Cf-4 and Cf-2. Comparison of the proteins co-purifying with Cf-4 and Cf-5 showed that the endogenous SOBIR1 protein pool of *N. benthamiana*, consisting of Niben101Scf03816g01001 (*Nb*SOBIR1a) and Niben101Scf04099g05004 (*Nb*SOBIR1b), was significantly enriched in the interactome of Cf-4 (Fig. 6b), whereas when Cf-4 and Cf-2 were compared, only Niben101Scf04099g05004 (*Nb*SOBIR1b) was significantly enriched (Fig. 6c). This observation supports our finding that the Cf-4 pool that is associated with SOBIR1, is indeed larger than the pool of Cf-2 and Cf-5 that is associated with SOBIR1.

### The amount of SOBIR1 protein interacting with the total pool of Cf protein is at least partially determined by their C-terminal tail

**Figure 7:**
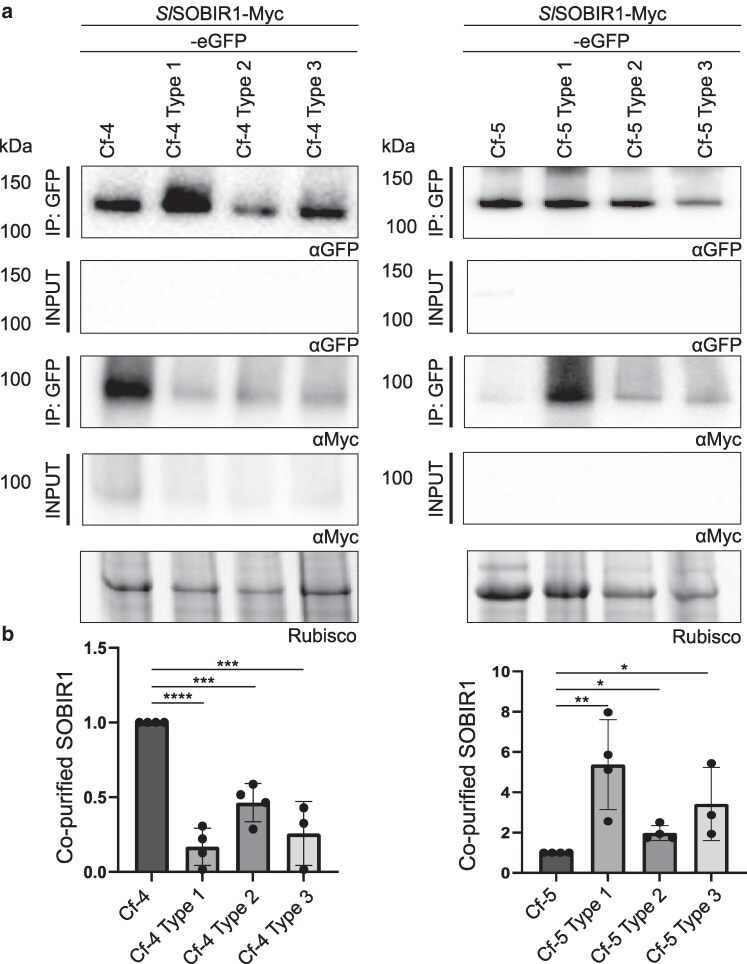
The C-terminal tail of Cf proteins plays a role in the affinity of the Cf protein for SOBIR1. a) eGFP-tagged Cf-4 and Cf-5 and the various chimeric Cf proteins were transiently co-expressed with Myc-fused *Sl*SOBIR1 in leaves of *N. benthamiana* (OD_600_ = 0.5). At 2 dpi, total protein was extracted and subjected to immunoprecipitation (IP) using GFP-trap beads, followed by western blotting with αGFP (upper panels) and αMyc (bottom panels) antibodies. b) Relative quantification of the amounts of *Sl*SOBIR1 protein co-purifying with Cf-4 and Cf-5 and with the chimeric Cf proteins. The relative amount of *Sl*SOBIR1 co-purifying with the wild-type Cf proteins was set to 1. An ANOVA/Dunnett's multiple comparison was used to determine statistical significance. Error bars display the SD. *P < 0.05; **P < 0.01; ***P < 0.001; ****P < 0.0001.

As shown above, Cf-5 and Cf-2 both trigger a weaker HR-related cell death and interact with lower amounts of SOBIR1 protein when compared to Cf-4 and Cf-9, which both trigger strong cell death and recruit larger amounts of SOBIR1 protein. The presence of a relatively large pool of Cf-4 and Cf-9 protein interacting with SOBIR1 might be related with this strong response. The C-terminal tail of Cf proteins might be involved in the strength of the interaction with SOBIR1, with a stronger interaction possibly leading to a larger SOBIR1-associated Cf protein pool, resulting in HR-related cell death of higher intensity. To test this hypothesis, we performed co-immunoprecipitation assays between SOBIR1 and Cf-4 and Cf-5, and also between SOBIR1 and the chimeric Cf-4 and Cf-5 proteins. The different Cf proteins, all tagged with eGFP, were co-expressed by agroinfiltration with *Sl*SOBIR1 tagged with Myc and at 2 dpi, total proteins were extracted. The eGFP-tagged Cf proteins were immunoprecipitated with GFP beads, and Cf-4, Cf-5, and the chimeric Cf proteins were visualized by incubating western blots with αGFP antibodies, while αMyc antibodies were used to visualize *Sl*SOBIR1. We observed that the chimeric Cf-5 proteins containing the C-terminal tail of Cf-4 (Type 1 and Type 2, Fig. 2a), giving strong HR-related cell death, indeed interacted with larger amounts of SOBIR1 protein than Cf-5 itself, which gives a less strong response. On the other hand, the chimeric Cf-4 proteins containing the C-terminal tail of Cf-5 (Type 1 and Type 2, Fig. 2a), giving less strong HR-related cell death, were found to interact with lower amounts of SOBIR1 protein than Cf-4 itself, which gives a strong response (Fig. 7).

This result indicates that the C-terminal tail of Cf proteins indeed plays a role in their interaction with SOBIR1. Moreover, the TM domain of Cf proteins also seems to have a role in the strength of the interaction with SOBIR1, as the Type 3 chimeric Cf-4 and Cf-5 proteins in which only the TM domains were swapped (Fig. 2a) also showed lowered and increased recruitment levels of SOBIR1, respectively (Fig. 7). However, the TM domainof Cf proteins did not show any involvement in determining the intensity of the HR-related cell death that is triggered by the chimeric Cf proteins (Fig. 2b and 2c), suggesting that a strong interaction between SOBIR1 and the Cf protein is not the only explanation for a more intense response.

## Discussion

### The cytoplasmic C-terminal tail of Cf proteins plays a role in determining the intensity of the HR-related cell death but is not involved in the initiation of early immune responses

In this study, we show that chimeric Cf-4 constructs containing the cytoplasmic C-terminal tail of Cf-5 trigger a much weaker HR-related cell death than Cf-4 itself and Cf-4 constructs only containing the Cf-5 transmembrane domain. Conversely, chimeric Cf-5 constructs having the Cf-4 cytoplasmic C-terminal tail induce a stronger response than wild-type Cf-5, and Cf-5 constructs only containing the Cf-4 transmembrane domain (Fig. 2), and this stronger cell death response is NRC2/3 dependent, like for Cf-4 itself (Figure S4). This observation indicates that the enhanced intensity of the HR-related cell death is mediated through the same downstream immune pathway rather than through activation of an alternative signaling pathway. The helper NLRs NRC2 and NRC3 oligomerize upon recognition of the effector (Contreras et al. 2023; Huang et al. 2024a), and it has been proposed that Cf-4/Avr4-mediated immunity might activate the NRC-dependent oligomerizations because of its requirement for NRC2 and NRC3 (Kourelis et al. 2022) Together, these observations suggest that Cf proteins signal through helper NLR oligomerization, and that the C-terminal tail of the Cf proteins modulates the strength of this immune response. A chimeric Cf-5/Cf-2 protein showed that the single amino acid difference between Cf-5 and Cf-2 in their C-terminal tail does not make a difference in determining the intensity of the cell death response (Figure S3). Additionally, all Cf-4 and chimeric Cf-4 proteins trigger a typical biphasic ROS burst, with no differences in the ROS burst intensity and downstream MAPK activation as detected by anti-p42/p44-ERK antibody at the 15-min time point, while Cf-5 and chimeric Cf-5 proteins showed similar results (Fig. 3). These findings together demonstrate that the cytoplasmic C-terminal tail of Cf proteins, rather than the transmembrane domain, has a specific role in determining the intensity of the HR-related cell death, but not in determining the intensity of the apoplastic ROS burst and MAPK activation as detected by the anti-p42/p44-ERK antibody.

The Cf-4/Avr4-triggered ROS burst, MAPK activation and cell death are differentially regulated by specific RLCK-VII family members, which illustrates that these immune responses are uncoupled (Huang et al. 2024b). Our study reveals that the C-terminal tail of Cf proteins specifically regulates the intensity of the HR-related cell death, without affecting the intensity of other immune responses. Basal activation of the intracellular KD of SOBIR1 by the C-terminal tail of Cf proteins might be sufficient for the ROS burst and MAPK activation at 15 min, while the cell death might require additional regulation of the intracellular KD of SOBIR by the C-terminal tail. This suggests that distinct activation mechanisms might exist for these immune responses.

### The C-terminal tail of Ve2 fails to initiate cell death and a ROS burst

It has been shown earlier that chimeric Ve1 proteins containing the transmembrane domain and cytoplasmic C-terminal tail of Ve2 failed to trigger HR-related cell death upon challenge with Ave1, leading to the conclusion that Ve2 has a cytoplasmic C-terminal tail that fails to trigger cell death (Fradin et al. 2014). In addition, our study showed that chimeric Cf proteins containing the cytoplasmic C-terminal tail of Ve1 successfully trigger cell death and a ROS burst upon recognition of their matching effector. However, chimeric Cf proteins containing the cytoplasmic C-terminal tail of Ve2 are unable to trigger either cell death or a ROS burst (Fig. 4). In addition, we observed that chimeric Cf proteins containing the cytoplasmic C-terminal tail of either Ve1 or Ve2 still interact with the co-receptors SOBIR1 and BAK1, proving that the incapability of chimeric Cf_Ve2 proteins to initiate downstream signaling is not due to a compromised interaction with the required co-receptors (Figure S5). It has been proposed that autophosphorylation of the KD of SOBIR1 as a result of SOBIR1 dimerization and transphosphorylation of the KD of BAK1 by SOBIR1 are required to initiate downstream signaling (van der Burgh et al. 2019; Huang et al. 2024b). Notably, the cytoplasmic C-terminal tail of Ve2 is much longer than that of Ve1 and any other RLP identified up till now. This unusually extended tail may affect the initiation of downstream signaling. How the C-terminal tail of Ve2 exactly interferes with the initiation of downstream signaling has yet to be determined.

### The C-terminal tail of Cf-4 is required for the initiation of HR-related cell death

Previous studies on RXEG1 and Ve1 have shown that a complete deletion of the cytoplasmic C-terminal tail abolishes the HR-related cell death upon recognition of their matching effector, showing that this tail is essential for the initiation of downstream signaling (Fradin et al. 2014; Sun et al. 2022). However, a complete deletion of the cytoplasmic part of an RLP might also disrupt their PM-associated stabilization, as it has been shown that the cytoplasmic tail of Toll-like receptors (TLRs) in animal systems contains amino acid residues that mediate binding to the PM (Kornilov et al. 2023). Although similar mechanisms have not been shown for RLPs in plants, for RLPs the cytoplasmic tail might also contribute to the association with the PM. For this, further truncation analysis of Cf-4 should be performed, guided by secondary structure predictions of the cytoplasmic C-terminal tail of Cf-4. To understand the requirements of the cytoplasmic C-terminal tail to trigger cell death, we generated a Cf-4 Δcoil truncation that resulted in a significant reduction of the intensity of the HR-related cell death when challenged with Avr4, whereas the α-helix truncation almost abolished the HR-related cell death (Fig. 5c). Furthermore, both truncations significantly decreased the ROS burst when compared to Cf-4 itself (Fig. 5d and 5e). These results indicate that the full-length C-terminal tail of Cf-4 is indeed required for proper initiation of downstream signaling. The C-terminal tail swapping strategy preserves the conserved overall structural features of the tail, including the predicted coil regions and α-helix. In contrast, truncations may disrupt the structural integrity of the C-terminal tail and may potentially affect receptor stabilization at the PM. Therefore, the approach of generating chimeric proteins provides a less structurally disruptive strategy to assess the contribution of the C-terminal tail to the HR-related cell death.

Interestingly, TLRs have an α-helix structure within their cytoplasmic C-terminal tail, and the adaptor proteins of TLRs interact with this tail via an α-helix structure (Patra et al. 2018; Patra and Choi 2018). Here, we observed that the α-helix truncation resulted in a more severe reduction of the intensity of the HR-related cell death than the Cf-4Δcoil truncation (Fig. 5), which suggests that, similar to TLRs, the α-helix within the cytoplasmic C-terminal tail of Cf-4 might be important for the interaction with its co-receptors and/or modulation of their kinase activity, although both truncated Cf-4 proteins retained the ability to interact with SOBIR1 (Figure S7).

### Both the transmembrane domain and C-terminal tail determine the affinity of Cf proteins for SOBIR1, but the C-terminal tail determines the intensity of the HR-related cell death

The strength of the interaction between RLPs and their co-receptor SOBIR1 appears to differ (Liebrand et al. 2013). From our observations, it has become clear that indeed the interaction of Cf-4 and Cf-9, which trigger a strong HR-related cell death, with SOBIR1 is stronger than the strength of the interaction of Cf-5 and Cf-2 with SOBIR1, which induces a weaker cell death, as the pool of SOBIR1-associated Cf protein is much larger for Cf-4 and Cf-9 (Fig. 6). Therefore, probably the observed differences between the SOBIR1-associated Cf-4 and Cf-9 pools and the SOBIR1-associated Cf-2 and Cf-5 pools reflect differences in the binding affinity of the various Cf proteins for SOBIR1. Furthermore, it has been shown that the transmembrane domain and cytoplasmic C-terminal tail of Cf-4 and Cf-9 are sufficient to recruit SOBIR1 (Wu et al. 2019). To understand whether both the cytoplasmic C-terminal tail and transmembrane domain contribute to the strength of the interaction with SOBIR1, and whether this strength positively correlates with the intensity of the HR-related cell death, we performed co-IP assays between SOBIR1 and the various chimeric Cf proteins. Cf-4 Type 3, which only contains the transmembrane domain of Cf-5, showed a reduced interaction with SOBIR1 when compared to Cf-4 itself. A similar reduction was observed for Cf-4 Type 2, which only contains the cytoplasmic C-terminal tail of Cf-5. Consistently, Cf-5 Type 3, only containing the transmembrane domain of Cf-4, showed an increased strength of the interaction with SOBIR1, whereas Cf-5 Type 2, only containing the transmembrane domain of Cf-4, exhibited a similar increase of this strength (Fig. 7). These results indicate that both the transmembrane domain and the cytoplasmic C-terminal tail of the Cf proteins contribute to the strength of their interaction with SOBIR1. Previous studies on the transmembrane domain of RLPs showed that this domain, which, for example, in various RLPs carries a GxxxG motif (Gust and Felix 2014), is essential for the interaction with SOBIR1 (Bi et al. 2016; Albert et al. 2019). The tail of Cf proteins contains relatively large numbers of basic amino acids, while the matching domain of SOBIR1 carries various acidic amino acids, suggesting an opposite charge-based interaction between them (Gust and Felix 2014; Bi et al. 2016; Snoeck et al. 2023) . Specifically, the transmembrane and C-terminal tail of Cf-4 together enhance the strength of its interaction with SOBIR1, whereas these domains from Cf-5 appear to reduce this strength. Although the transmembrane domain of Cf proteins contributes to the strength of the interaction, there is no evidence that this domain has a role in determining the intensity of the HR-related cell death. In contrast, the C-terminal tail of the Cf proteins not only contributes to the strength of the interaction with SOBIR1, but is also involved in determining the strength of the response, possibly through additional modulation of the kinase activity of SOBIR1 (Fig. 2).

It has been shown that co-expression of *At*RLP30 with *At*SOBIR1 triggers a stronger ethylene response than its co-expression with *Nb*SOBIR1 or *Sl*SOBIR1. It was also reported that RLP23_Cf-9 chimeric proteins interact more strongly with *Sl*SOBIR1, as compared to RLP23 itself. Therefore, it was proposed that there is a species-specific interaction between RLPs and SOBIR1 (Snoeck et al. 2023; Yang et al. 2023, 2025). We show that although all Cf proteins are tomato RLPs, their interaction with SOBIR1 varies within the same species (Fig. 6). Cf proteins need SOBIR1 for their stabilization and even proper accumulation on the PM (Liebrand et al. 2013; Gust and Felix 2014; Bi et al. 2016). Moreover, the variation in the affinity of the various Cf proteins for *Sl*SOBIR1 may affect the activity of the individual Cf proteins that might be stacked in tomato, which might affect the resistance against different strains of *F. fulva* expressing the matching effectors.

Wu et al. (2019), showed that transgenic tobacco plants expressing an EFR_Cf-4/9 chimeric protein provide enhanced resistance against (hemi)biotrophic bacterial pathogens, without any morphological changes in the tobacco leaves. In addition, Yang et al. (2025), reported that tomato plants expressing RLP23_Cf-9 or RLP23_EIX2 show enhanced, broad-spectrum disease resistance when compared to wild-type tomato and tomato lines expressing RLP23. However, Cf-4 and Cf-5 do provide full resistance against *F. fulva* producing the matching Avrs, and it was even reported that Cf-5 provides a stronger resistance to the fungus than Cf-4 (Hammond-Kosack 1994). It would be interesting to investigate whether the chimeric Cf proteins provide different levels of resistance against *F. fulva*.

BAK1 is a common regulatory co-receptor for RLPs and RLKs that are involved in growth, development and immunity. While the kinase activity of BAK1 is shared across different RLP/RLK complexes and RLKs, the downstream signaling output varies depending on the primary receptor itself (Chinchilla et al. 2009; Ma et al. 2016). Studies on chimeric RLKs acting as primary receptors and of which the function is dependent on BAK1, revealed that the specificity of the signaling output is determined by the intracellular KD of the receptor, rather than by BAK1, and provide experimental evidence that BAK1 is a neutral co-receptor across all RLKs acting as ligand receptors and only required for their trans-activation by phosphorylation (Hohmann et al. 2020). In the chimeric proteins generated in this study, the ID and the last 4 LRRs, which are regions previously shown to be important for BAK1 recruitment, were conserved. Therefore, we propose that in Cf/SOBIR1 receptor complexes, BAK1 functions as a neutral co-receptor that facilitates receptor activation but does not determine the intensity of the HR-related cell death. Overall, our results show that the C-terminal tail of RLPs determines the intensity of the cell death response and contributes to the strength of the interaction with SOBIR1. However, a stronger interaction with SOBIR1 does not always correlate with a stronger response, as revealed by the Cf chimeras containing only the transmembrane domain of another Cf protein (Fig. 2). Interestingly, while the C-terminal tail of Cf proteins does not contribute to the intensity of the ROS and MAPK activation, its partial deletion does affect both the apoplastic ROS and the HR-related cell death (Fig. 5). We propose that the C-terminal tail plays a specific role in promoting SOBIR1 function during immune signaling, and that a full-length tail is required for this process. Furthermore, the length and amino acid composition of the tail might eventually determine the actual intensity of the HR-related cell death. Overall, note that a limitation of this study is that all experiments were performed using transient expression of the constructs in leaves of *N. benthamiana*. Although the various Cf proteins and their chimeras were detected at similar levels upon GFP immunoprecipitation, possible differences in the original protein expression levels cannot be excluded.

## Materials and methods

### Plant maintenance conditions

*N. benthamiana* plants were grown in a controlled climate chamber under a 16-h light cycle at 25 °C, an 8-h dark cycle at 21 °C, and at 70% humidity.

### Generation of the vectors for agrobacterium transformation

The *Cf-4*, *Cf-5*, *Cf-2*, *Ve1*, and *Ve2* genes were described previously (Dixon et al. 1996; Thomas et al. 1997; Dixon et al. 1998; Kawchuk et al. 2001). Plasmids containing the open reading frames (ORFs) of these genes were used as templates to amplify their coding sequences with Phusion Hot Start II High-Fidelity Polymerase (Promega) and specifically designed primers, of which the sequences are listed in Table S1. The amplified coding sequences were subsequently ligated into the pENTR/D-TOPO vector. pENT plasmids served as a backbone to generate the various chimeric constructs, using the primers of which the sequences are listed in Table S1. The chimeric constructs were inserted into the pENTR/D-TOPO vector by the ClonExpress MultiS One Step Cloning Kit. The coding sequences of all constructs were then transferred to the pBIN-35S binary vector using a Gateway LR Clonase II Enzyme mix (Thermo Fisher Scientific). In this way, the constructs referred to as “pBIN-35S::,” and all introducing a C-terminal eGFP tag, were obtained; pBIN-35S::Cf-5 (SOL10519), pBIN-35S::Cf-5 Type 1 (SOL10516), pBIN-35S::Cf-5 Type 2 (SOL10517), pBIN-35S::Cf-5 Type 3 (SOL10522), pBIN-35S::Cf-4 (SOL10900), pBIN-35S::Cf-4 Type 1 (SOL10901), pBIN-35S::Cf-4 Type 2 (SOL10902), pBIN-35S::Cf-4 Type 3 (SOL10903), pBIN-35S::Cf-2 (SOL10533), pBIN-35S::Cf-5_Cf-2 (SOL10542), pBIN-35S::Cf-5_Ve1 (SOL10547), pBIN-35S::Cf-5_Ve2 (SOL10544), pBIN-35S::Cf-4_Ve1 (SOL10548), pBIN-35S::Cf-4_Ve2 (SOL10545), pBIN-35S::Cf-2_Ve1 (SOL10549), and pBIN-35S::Cf-2_Ve2 (SOL10546).

NetSurfP-3.0 was used to predict the secondary structure of the C-terminal tails (https://services.healthtech.dtu.dk/services/NetSurfP-3.0/). The pENT-Cf-4 plasmid was used as a backbone to generate the 2 deletion constructs of the C-terminal tail of Cf-4. For generating pENT-Cf-4Δα-helix, the insert of the pENTCf-4 plasmid was amplified with excluding the sequence encoding the α-helix domain by Phusion Hot Start II High-Fidelity Polymerase (Promega), while for generating the pENT-Cf-4Δcoil construct, the sequence encoding the coil region was excluded upon amplification. The sequences of the used primers are listed in Table S1. The constructs were transferred to the pBIN-35S binary vector by a Gateway LR Clonase II Enzyme reaction (Thermo Fisher Scientific), and the generated constructs are referred to as pBIN-35S::Cf-4Δα-helix (SOL10577) and pBIN-35S::Cf-4Δcoil (SOL10578). Constructs containing *Sl*SOBIR1-Myc (SOL2754) and *Sl*BAK1-Myc (SOL2555) were described previously (Liebrand et al. 2013; Postma et al. 2016).

### Production of the Avr5 protein in *Pichia pastoris*

Recombinant His-tagged Avr5 protein production was performed in the yeast *Pichia pastoris* (van den Burg et al. 2001). The culture supernatant was concentrated using a VIVAFLOW 200 device, with a 5,000 molecular weight cutoff (MWCO) membrane (Sartorius), at 4 °C. The concentrated supernatant was then dialyzed against equilibration buffer (25 mM Tris, 25 mM NaCl, 10 mM imidazole, pH = 7.5), using Spectrum Labs Spectra/Por 7 Pretreated Dialysis Tubing (Spectrum Labs; MWCO 3.5 kDa). The Avr5 protein was subsequently purified using a BioLogic LP low-pressure chromatography system (Bio-Rad), with a column containing His60 nickel beads Ni-NTA resin for purification of His-tagged proteins (Takara Bio). A 10- to 200-mM gradient of imidazole in elution buffer (25 mM Tris, 25 mM NaCl, pH = 7.5), over a period of 20 min at a flow of 1 mL/min, was used to elute the Avr5 protein from the column, after which the solution of purified Avr5 protein was dialyzed against MQ. Determination of the Avr5 protein concentration was performed by the Quick Start Bradford Protein Assay (Bio-Rad), and the presence of the protein was verified by immunoblotting using 6x-His Tag Monoclonal Antibody (Invitrogen, MA1-21315).

### HR-related cell death assay

For the HR-related cell death assays, we performed transient Agrobacterium-mediated transformation of the various constructs that were generated in leaves of *N. benthamiana*, as previously described (van der Hoorn et al. 2000). For this, the constructs were transiently expressed by agroinfiltration with their matching effectors in fully expanded leaves of 3- to 4-wk-old *N. benthamiana* plants (OD_600_ = 0.8, for all constructs), and for most constructs, the leaves were harvested and analyzed 5 d later. To quantify the intensity of the HR-related cell death, leaves were imaged by using the red-light imaging method with a Chemidoc XRS system (Bio-Rad) (Landeo Villanueva et al. 2021).

### Apoplastic ROS burst assay

To determine the intensity of the apoplastic ROS burst, constructs were individually expressed by agroinfiltration in fully expanded leaves of 3- to 4-wk-old *N. benthamiana* plants (OD_600_ = 0.1) and at 2 dpi, 5-mm leaf discs were taken from the leaves and incubated in 50 μL of MQ in a 96-well plate in the dark, at room temperature overnight. Next, the MQ was replaced by 50 μL of fresh MQ, and the leaf discs were incubated for another hour. Then, 50 μL of a reaction solution containing 50 μM luminol L-012 (FUJIFILM), 10 μg horseradish peroxidase, and either Avr4 protein, Avr5 protein, or elf18 peptide, at a concentration of 0.1 μM, was added to the wells. The apoplastic ROS burst was subsequently measured over a period of 5 h using a CLARIOstar plate reader (BMG LABTECH).

### MAPK activation assay

Cf-5-eGFP, Cf-4-eGFP, and their various chimeric constructs were transiently expressed in fully expanded leaves of *N. benthamiana* (OD_600_ = 0.8), and at 2 dpi, leaves expressing Cf-5 and the chimeric Cf-5 proteins were treated with a 5-µM solution of Avr5 protein, while leaves expressing Cf-4 and the chimeric Cf-4 proteins were treated with a 5-µM solution of Avr4. After 15 min, the leaves were harvested, and total proteins were extracted and separated by SDS–PAGE. Subsequently, the proteins were subjected to immunoblotting using anti-p42/p44-erk antibodies (NEB) to detect activated MAPKs.

### Immunoprecipitation and co-immunoprecipitation of eGFP- and Myc-tagged proteins

Immunoprecipitation (IP) and co-immunoprecipitation (co-IP) were performed as previously described (Liebrand et al. 2013). Proteins tagged with eGFP were detected using αGFP-HRP antibodies (Miltenyi Biotec, 130-091-833), while αMyc antibodies (cMyc9E10, sc-40, Santa Cruz) were used to detect Myc-tagged proteins, followed by incubation with αMouse-HRP (GE Healthcare) as a secondary antibody.

### LC-MS/MS analysis of proteins co-purifying with Cf proteins

Mass spectrometry (MS) analysis was performed to analyze the proteins co-purifying with Cf-4, Cf-5, and Cf-2. For this, the Cf proteins, all tagged with eGFP, were transiently expressed in fully expanded leaves of *N. benthamiana* (OD_600_ = 0.5), and at 2 dpi, the leaves were harvested, and total proteins were extracted as described previously (Liebrand et al. 2013). The total protein extract was incubated with 50 µL of GFP-trap magnetic agarose beads (Chromotek), in a rotary shaker with 10 rpm rotations per min, at 4 ˚C for 1 h. Then, the beads were pelleted by using a magnetic rack, washed 3 times with extraction buffer (50 mM Tris, 150 mM NaCl, containing 1 tablet of protease inhibitor cocktail [Sigma] per 50 mL, pH = 8), and then the beads were washed 3 times with ABC buffer (50 mM ammonium bicarbonate, pH = 8). Lastly, the beads were kept in 45 µL ABC buffer, and the beads were incubated with 5 µL dithiothreitol (DTT) (150 mM), at 45 ˚C for 30 min to reduce the disulfide bonds present in the captured proteins. Next, 6 µL of acrylamide (200 mM) was added, after which the beads were incubated at room temperature for 10 min to alkylate the sulfhydryl groups of the captured proteins. The proteins were then released from the beads by a tryptic digestion. For this, the beads were incubated overnight with 100 µL of a trypsin solution (50 ng/µL in ABC buffer), at room temperature with mild agitation. After overnight incubation, the activity of the trypsin enzyme was stopped by acidification (pH = 3) of the samples with trifluoroacetic acid. Lastly, the peptides were cleaned up using µColumns (Wendrich et al. 2017).

The peptide analyses were performed by NanoLC-MS/MS, using a Thermo Vanquish Neo nanoLC system coupled to a Thermo Orbitrap Exploris 480 mass spectrometer (Thermo Fisher Scientific, Bremen, Germany), as described before (Zheng et al. 2024). The proteins were identified by using the Andromeda search engine in MaxQuant, with LFQ, and the *N*. *benthamiana* proteome database (Niben 1.0.1). The protein sequences of Cf-4, Cf-5, and Cf-2, as well as the general contaminants dataset, were added as separate databases. MaxQuant false discovery rates were set to 0.01 on peptide and protein levels, and the analysis was performed as described previously (Smaczniak et al. 2012; Wendrich et al. 2017).

The identified proteins were analyzed by using Perseus (Tyanova et al. 2016), and potential contaminants and reverse proteins were removed from the dataset. Proteins identified less than 3 times across the 3 replicates were also filtered out. The LFQ intensity values were transformed using a log_2_ scale, and the missing values were replaced from a normal distribution. A 2-sample test was performed to compare 2 different treatments, and the results were represented by volcano plots, using a permutation-based adjustment (FDR = 0.005, 250 randomizations, with the S0 set to 0.1).

## Supplementary Material

kiag476_Supplementary_Data

## Data Availability

The data underlying this article are available in the article and in its online supplementary material.
