# Supplementary material for: The C-terminal tail of Cf resistance proteins determines the intensity of the effector-triggered hypersensitive response-related cell death
Source: Plant Physiol. 2026 Jul 31;201(3):kiag476. doi: 10.1093/plphys/kiag476 (PMC13425104; doi:10.1093/plphys/kiag476)
Supplement: kiag476_Supplementary_Data [file kiag476_supplementary_data.zip › Supplementary Figures Final.docx]

**Supplementary Figure S1.** Cf-9, Cf-4, Cf-2/Rcr3, and Cf-5, all fused to eGFP, were transiently co-expressed in N. benthamiana (OD_600_=0.8) with their corresponding effectors Avr9, Avr4, Avr2, and Avr5, respectively. To test for specificity, Cf-9, Cf-4, and Cf-2 were also co-expressed with the non-matching effector Avr5, while Cf-5 was co-expressed with the non-matching effector Avr4, as a negative control. The leaves were imaged at 6 dpi by red light.

**Supplementary Figure S2.** Cf-4, Cf-5 and the chimeric Cf proteins accumulate at similar levels. Cf-4, Cf-5 and the chimeric Cf proteins, all fused to eGFP, were transiently expressed in *N. benthamiana* (OD_600_=0.5). At 2 dpi, total protein was extracted and subjected to immunoprecipitation (IP) using GFP-trap beads, followed by western blotting (WB) with αGFP antibodies (upper panels). Total protein loading is represented by Rubisco (lower panels).

**Supplementary Figure S3.** A chimeric Cf-5 protein containing the C-terminal tail of Cf-2 triggers HR-related cell death with an intensity similar to Cf-5 itself. **A)** Overview of the domain structure of the chimeric Cf protein that has been generated. The domains originating from the Cf-5 and Cf-2 proteins are indicated in yellow and green, respectively. **B and C**) Cf-5 and the chimeric Cf-5 protein, both fused to eGFP, were transiently co-expressed with their matching effector Avr5 in *N. benthamiana* (OD_600_=0.8). In **B** agroinfiltration of the right part of the leaves was performed with Cf-5/Avr5, whereas on the left the Cf-5_Cf-2-chimera was agroinfiltrated in combination with Avr5. The leaves were imaged at 6 dpi by visible (left) and by red light (right). **C)** The intensity of the HR-related cell death triggered by the Cf-5 and Cf-5_Cf-2 chimeric proteins upon challenge with their matching effector Avr5 was quantified by Image Lab. A Student’s *t*-test was used to determine statistical significance (unpaired two-tailed *t*-test, P < 0.05). Central line, median; error bars, min–max, n=5-6. ns: not significant.

**Supplementary Figure S4.** HR-related cell death triggered by the chimeric Cf-4 and Cf-5 proteins remains dependent on NRC2/3. **A and B**) Cf-4, Cf-4 Type 3, Cf-5 Type 1, and Cf-5 Type 2, all fused to eGFP, were transiently co-expressed with their matching effector in the leaf halves of wild-type (WT) *N. benthamiana* and in the NRC2/3 knock-out mutant *nrc2/3_3.3.1* (OD_600_=0.8). **A)** The leaves were imaged at 6 dpi by red light. **B)** The intensity of the HR triggered by Cf-4 and the chimeric Cf proteins upon challenge with their matching effector was quantified by Image Lab. A Student’s *t*-test was used to determine statistical significance (unpaired two-tailed *t*-test, P < 0.05). Central line, median; error bars, min–max n=5-6. The experiment was repeated three times, with each independent replicate indicated by a different color. **p < 0.01; ***p < 0.001.

**Supplementary Figure S5.** Chimeric Cf proteins containing the C-terminal tail of Ve1 or Ve2 properly interact with *Sl*SOBIR1 and *Sl*BAK1. Chimeric Cf proteins containing the C-terminal tail of Ve1 or Ve2, all fused to eGFP, were transiently co-expressed with either Myc-fused *Sl*SOBIR1 **(A)** or *Sl*BAK1 **(B)** in *N. benthamiana* (OD_600_=0.5). At 2 dpi, total protein extracts were made and subjected to immunoprecipitation (IP) using GFP-trap beads, followed by western blotting with αGFP (upper panels) and αMyc (middle panels). Total protein loading is represented by Rubisco (lower panels).

**Supplementary Figure S6.** Secondary structure of the C-terminal tail of Cf-4/9, Cf-2, Cf-5, Ve1 and Ve2, containing domains with a non-regular secondary structure (coil), an α-helix and β-sheets. **A)** The secondary structures of the C-terminal tails as predicted by NetSurfP - 3.0. The coil is shown as a pink line, α-helix domains are shown in orange, and β-sheets are indicated as blue arrows. **B)** AlphaFold structural models of the C-terminal tails were generated and the five conformations with the highest-probability were overlaid. The model confidence is color-coded, ranging from cyan (low confidence) to magenta (high confidence). Scale bar: 1 nm.

**Supplementary Figure S7.** Cf-4, and Cf-4 mutants of which parts of the C-terminal tail have been deleted, properly accumulate and constitutively interact with SOBIR1. Cf-4, Cf-4∆coil and Cf-4∆α-helix, all fused to eGFP, were transiently co-expressed with Myc-fused *Sl*SOBIR1 in *N. benthamiana* (OD_600_=0.5). At 2 dpi, total protein extracts were subjected to immunoprecipitation using GFP-trap beads, followed by western blotting (WB) with αGFP (upper panel) and αMyc (middle panel). Total protein loading is represented by Rubisco (lower panel).
