# Supplementary material for: The C-terminal tail of Cf resistance proteins determines the intensity of the effector-triggered hypersensitive response-related cell death
Source: Plant Physiol. 2026 Jul 31;201(3):kiag476. doi: 10.1093/plphys/kiag476 (PMC13425104; doi:10.1093/plphys/kiag476)
Supplement: kiag476_Supplementary_Data [file kiag476_supplementary_data.zip › Supplementary Table S1.docx]

**Supplementary Table S1:** Nucleotide sequences of the primers used in this study.

| Primer code | Primer name | Sequence (5′ - 3′) |
| --- | --- | --- |
| Eo_029 | pENT Fw* | AAGGGTGGGCGCGCCG^†^ |
| Eo_040 | Cf-5 TOPO Fw | CACCATGGGATTTGTTCTCTTTTCACA |
| Eo_041 | Cf-5 TOPO Rv | GAACCTATTGTTCCTCCGCC |
| Eo_045 | Lin. Cf-5 Type 1 Rv | GAAGTCGTTGAAGAACTCGGAGTTAGACT |
| Eo_046 | Insert for Cf-5 Type 1 Fw | ccgagttcttcaacgacttcTGGCAGGGGGTTCTCGT |
| Eo_047 | Insert for Cf-5 Type 1 Rv | gggtcggcgcgcccacccttATATCTTTTCTTGTGCTTTTTCATTTTC |
| Eo_048 | Lin. Cf-5 Type 2 Rv | GGATGTAGATGATGCTGATGCCG |
| Eo_049 | Insert for Cf-5 Type 2 Fw | tcagcatcatctacatcctgTGGTCAACTCAATATCCAGCATG |
| Eo_082 | Lin. Cf-5 Type 3 Fw | ATCAGCACCGGGAACCTTAGA |
| Eo_083 | Insert Cf-9 TM Fw | ccgagttcttcaacgacttcTGGCAGGGGGTTCTCGTG |
| Eo_084 | Insert Cf-9 TM Rv | ctaaggttcccggtgctgatCATTATGTATATTACGGACAGTCCAATAA |
| Eo_104 | Insert Cf-2 for Cf-5 Fw | tcagcatcatctacatcctgATCTCTACTGGAAACCTTAGATGGCT |
| Eo_105 | Insert Cf-2 for Cf-5 Rv | gggtcggcgcgcccacccttGAACCTGTTGTTTCTTCTTCTGTAGTTC |
| Eo_110 | Lin. pENT_Cf-2 Rv | AAGGATGTAGATGATAGAGATTCCGA |
| Eo_111 | Insert Ve2 for Cf-5 Fw | tcagcatcatctacatcctgTACAAGCAAGGAAACAAATACTTTGA |
| Eo_112 | Insert Ve2 for Cf-5 Rv | gggtcggcgcgcccacccttAAACTTTTTGTGATATATGACTAATAAAGGTG |
| Eo_113 | Insert Ve2 for Cf-4 Fw | tgtccgtaatatacataatgTACAAGCAAGGAAACAAATACTTTGA |
| Eo_112 | Insert Ve2 for Cf-4 Rv | gggtcggcgcgcccacccttAAACTTTTTGTGATATATGACTAATAAAGGTG |
| Eo_114 | Insert Ve2 for Cf-2 Fw | tctctatcatctacatccttTACAAGCAAGGAAACAAATACTTTGA |
| Eo_112 | Insert Ve2 for Cf-2 Rv | gggtcggcgcgcccacccttAAACTTTTTGTGATATATGACTAATAAAGGTG |
| Eo_116 | Ve1 for Cf-5 Fw | tcagcatcatctacatcctgTACAAGCCAGTGAAGAAATGGTTT |
| Eo_117 | Ve1 for all Cf Rv | gggtcggcgcgcccacccttCTTTCTTGAAAACCAAAGCAAGC |
| Eo_118 | Ve1 for Cf-4 Fw | tgtccgtaatatacataatgTACAAGCCAGTGAAGAAATGGTTT |
| Eo_119 | Ve1 for Cf-2 Fw | tctctatcatctacatccttTACAAGCCAGTGAAGAAATGGTTT |
| Lo1 | Lin. Cf-4 wo TM Fw | TGGTCAACTCAATATCCAGCATGG |
| Lo2 | Lin Cf-4 wo TM Rv | ACTGATCATTGGTGAATCTTCTTCCTCC |
| Lo3 | Lin. Cf-4 wo TM_iJM Fw | AAGGGTGGGCGCGCCG |
| Lo4 | Lin. Cf-4 wo iJM Rv | CATTATGTATATTACGGACAGTCCAATAACAAGTCC |
| Lo5 | Insert Cf-5 TM Fw | aagattcaccaatgatcagtTGGAAGGCTGCACTGATGGG |
| Lo6 | Insert Cf-5 TM Rv | gctggatattgagttgaccaCAGGATGTAGATGATGCTGATGCC |
| Lo7 | Insert Cf-5 TM-iJM Rv | gggtcggcgcgcccacccttGAACCTATTGTTCCTCCGCCG |
| Lo8 | Insert Cf-5 iJM Fw | tgtccgtaatatacataatgATCAGCACCGGGAACCTTAGA |

^*^Fw, forward; Rv, reverse.

^†^Sequences that overhang and match with the pENT vectors are shown in lowercase letters.
